# The role of hypoxia-inducible factors in neovascular age-related macular degeneration: a gene therapy perspective

**DOI:** 10.1007/s00018-019-03422-9

**Published:** 2019-12-31

**Authors:** Parviz Mammadzada, Pablo M. Corredoira, Helder André

**Affiliations:** Division of Eye and Vision, Department of Clinical Neuroscience, Karolinska Institutet, St. Erik Eye Hospital, Stockholm, Sweden

**Keywords:** Age-related macular degeneration, Angiogenesis, Hypoxia-inducible factors, Gene therapy

## Abstract

Understanding the mechanisms that underlie age-related macular degeneration (AMD) has led to the identification of key molecules. Hypoxia-inducible transcription factors (HIFs) have been associated with choroidal neovascularization and the progression of AMD into the neovascular clinical phenotype (nAMD). HIFs regulate the expression of multiple growth factors and cytokines involved in angiogenesis and inflammation, hallmarks of nAMD. This knowledge has propelled the development of a new group of therapeutic strategies focused on gene therapy. The present review provides an update on current gene therapies in ocular angiogenesis, particularly nAMD, from both basic and clinical perspectives.

## Introduction

Age-related macular degeneration (AMD) is the most common cause of visual loss in developed countries, with particular incidence in the geriatric population [1]. AMD is the third global cause of blindness (8.7% of patients’ blindness), preceded by cataract and glaucoma [2]. By the year 2020, AMD patients are predicted at 196 million, rising to 288 million in 2040, in association with the global aging of human population [3]. Patients with AMD suffer from impaired fine and color vision, with particular clinical relevance when the fovea (center of the macula, containing the highest density of cone photoreceptors) is affected and the central field of vision is compromised [4, 5].

Cellular events in AMD involve disruption of photoreceptors, retinal pigment epithelial (RPE) cells, Bruch’s membrane (BM), and the choroid. The observed cellular events are a result of destabilized homeostasis of reactive oxygen species (ROS) response [6], phagocytosis [7, 8], extracellular matrix remodeling [9], and alternative complement-related inflammation [10].

Etiologically, AMD is a multifactorial disease. Genetic variants associated with AMD include complement factor (CF)H [11] and CFH-related genes 1 to 5 [12], complement protein (C)3 [13], C9 [14], age-related maculopathy susceptibility (ARMS)2 gene [15], and the vascular endothelial growth factor (VEGF) and VEGF receptor (VEGFR) axis [16, 17]. In addition, other genetic variants have shown a causal link to AMD, such as tissue inhibitor metalloproteinase (TIMP) 3 [18], fibrillin [19], collagen 4A3, and metalloproteinase (MMP) 19 and − 9 [20]. Moreover, environmental factors, e.g., advanced age, female gender, white race, smoking, increased body mass index, hypertension, and hyperopia, have been suggested to predispose to AMD [21]. In addition, certain biomarkers as carboxyethylpyrrole [22] and homocysteine [23, 24] have been shown to correlate with AMD. Despite this knowledge, AMD etiology is still evasive, urging a need for further research to deepen the understanding of AMD initiation and progression.

Here, the role of hypoxia and the hypoxia-inducible factors (HIFs) are revised in a perspective of AMD initiation and progression, as well as putative therapeutic targets, as they are the predominant molecular pathways of neovascular AMD (nAMD) [25–29].

## Types of AMD

In general terms, AMD is designated according to disease progression and symptoms into early, intermediate, and advanced AMD [30]. Advanced cases (3% of the population [31]) are categorized into two types: “dry” or geographic atrophic form, and “wet” or neovascular form [30]. Atrophic AMD (aAMD) is more common but spanning only 20% of greater central visual loss. nAMD comprehends fewer cases, but has been related with 80% of greater central vision loss [32–34]. Albeit not a clinical constant, some cases of aAMD progress into nAMD, illustrating a possible clinical evolution or a certain redundancy between the two advanced types of AMD [4, 35, 36].

### Drusen maculopathy

A common characteristic of AMD is pigment alteration in the RPE and hard or soft extracellular deposits between the RPE and the BM, referred to as drusen deposits [5, 37–39]. Soft drusen have been associated with higher risk of developing advanced AMD [40] and involved in the progression into geographic atrophy and causally associated with the initiation of choroidal neovascularization (CNV). CNV results in the formation of immature and fragile blood vessels that cause exudates into the subretinal spaces [41]. In rare cases, large soft drusen assemble and form pigment epithelial detachments (PEDs), which lead to an elevation of the RPE under the retina and impair the oxygenation of RPE cells, physiologically dependent on the intricate contact with the choroidal vascular bed [42].

The Beaver Dam Eye Study estimates that the prevalence of drusen is 2% in persons 43–54 years of age and 24% in persons over 75 years of age [43, 44]. Drusen are accepted as one of the causes of AMD, but the molecular pathway of drusen formation is still elusive [5, 37–39].

Drusen deposits are understood as the result of an incomplete processing of photoreceptor outer segments (POS) by the RPE [45], which cannot transmigrate through the BM for removal by the choriocapillaries. Such deposits create a barrier that decreases the diffusion of oxygen and nutrients from the choroid to the photoreceptors and the RPE in one direction, and decreases the removal of debris by the choriocapillaries into the other direction [4, 5, 37, 46]. Drusen contain lipids [47, 48], glycoconjugates [49, 50], glycosaminoglycan [51], and pigments, such as lipofuscin [52]. Concomitantly, the presence of advanced glycation end-products in drusen and on the BM, a product of aging, seems to contribute to AMD pathogenesis [53]. Finally, when cholesterol accumulates, soft drusen are formed [54, 55] and a higher risk association for advanced AMD is denoted. Drusen proteomic analysis has demonstrated the presence of multiple proteins, among which TIMP3 [18], clusterin, vitronectin, and serum albumin were the most common proteins in normal donor drusen, whereas crystallins were more frequently present in AMD donor drusen [38, 56, 57]. Drusen inflammatory and complement proteins, such as beta amyloid [58], immunoglobulin light chains, factor X, and C5 and − 5b, have received special attention [59], specifically since CFH [11] and -B [60] gene mutations have been associated with AMD [61].

Lipofuscin [52], an aging pigment containing the autofluorescent oxidatively modified lipid *N*-retinylidene-*N*-retinylethanolamine (A2E) [62, 63] together with minimal amounts of oxidatively modified proteins [64], is the product of impaired phagocytosis of the POS due to decreased RPE lysosomal activity [65]. Physiologically, lipofuscin is degraded completely or exocytosed by the RPE cells basolaterally and removed by the choroidal blood stream [66]. In AMD, lipofuscin accumulates intracellularly in the RPE and on the BM and is highly present in drusen deposits [67]. In addition, age-dependent reduction in enzyme and mitochondria functions results in ROS accumulation, which contributes to the pathogenesis of AMD [6, 8].

**Fig. 1:**
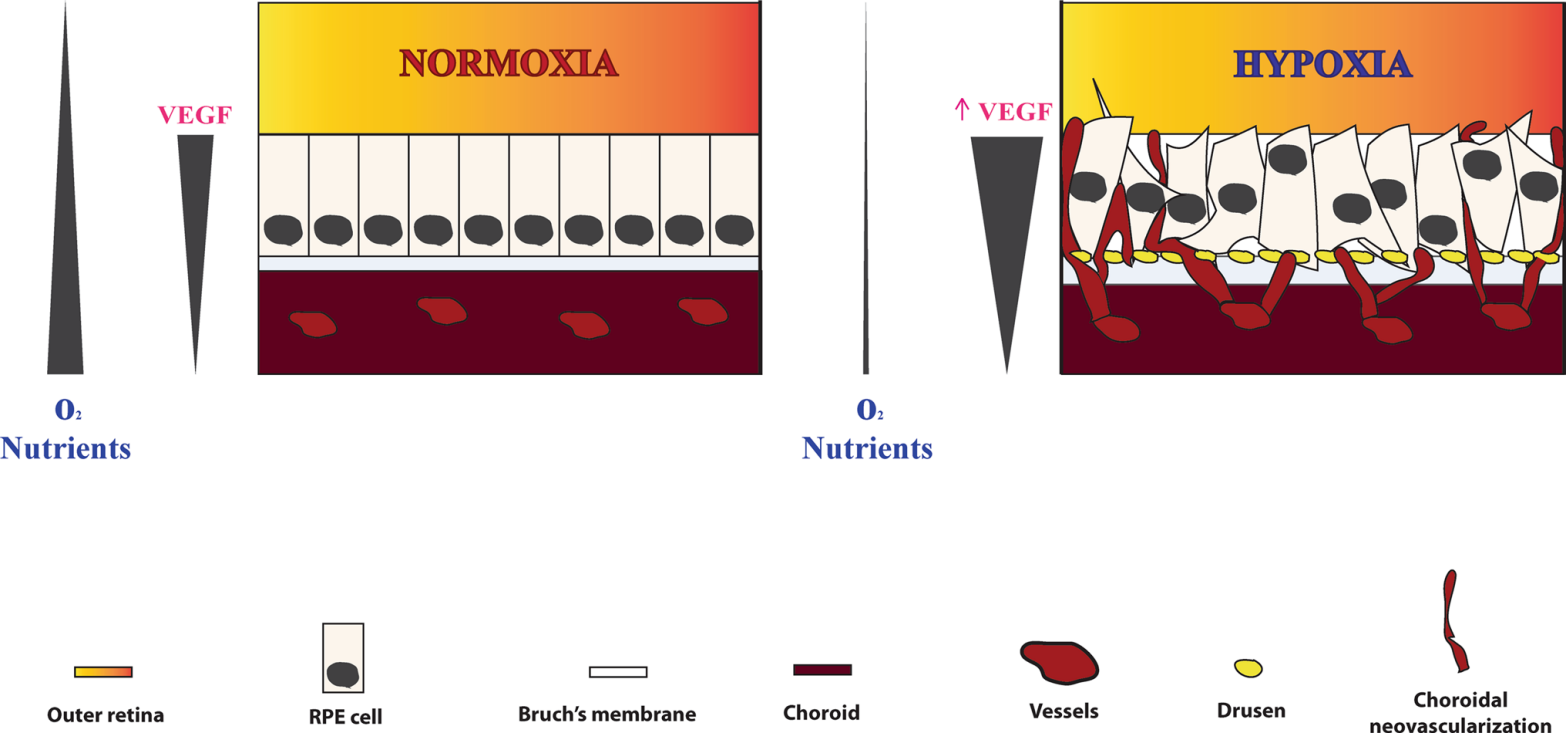
Schematic representation of nAMD pathogenesis. **a** In physiologic conditions, a flow of oxygen and nutrients raises from the choroid to the outer retina. The RPE produces physiologic levels of VEGF to sustain the proximity of choroidal vessels and maintenance of a normoxic status. **b** In early AMD, the disruption of several cellular mechanisms damages RPE cells and results in the formation of sub-RPE drusen deposits and thickening of Bruch’s membrane. These events create a relative hypoxic condition in the outer retina, due to decreased oxygen and nutrients flow from the distanced choroid. In response, RPE cells upregulate production of VEGF, which leads to choroidal neovascularization and ultimately a dystrophic RPE layer

In sum, drusen maculopathy results from the combination of accumulated lipofuscin, ROS, and complement alternative-related inflammation, and the decreased extracellular matrix remodeling and phagocytosis. These culminate in the thickening of Bruch’s membrane, creating a barrier that reduces the diffusion of oxygen and nutrients from the choroid to the photoreceptors and the RPE, and decreases the removal of debris by the choriocapillaries [4, 5, 37, 46]. Ultimately, macular drusenopathy leads to a status of relative hypoxia within the subretinal compartments and contributes to the pathogenesis of AMD (Fig. 1) [25, 68, 69]. In response, the RPE is triggered to secrete proangiogenic factors, such as VEGF into the choroidal space, in an attempt to minimize hypoxia-induced events. Hypoxia stimulates secretion of not only VEGF but several vasculogenic and inflammatory cytokines by the RPE, thus contributing to the development of CNV [70–73].

### Treatment of AMD

To date, there is neither an effective preventive treatment [74–76] nor a cure for AMD. Yet if untreated, AMD will evolve to a severe and irreversible disciform scar, resulting in extremely compromised vision or even blindness [4, 5].

Nowadays, nAMD is treated by routine intraocular injections of anti-VEGF agents [77–83], and to some extent by photodynamic therapy and thermal laser [84, 85]. The use of intravitreal anti-VEGF agents has revolutionized the treatment of nAMD with pegaptanib being the first approved anti-VEGF agent. Since pegaptanib binds to a single isoform of VEGF, it showed less efficacy. Later bevacizumab and ranibizumab have been introduced as anti-VEGF agents in the treatment of nAMD. Both these molecules bind all isoforms of VEGF. In comparison with bevacizumab, ranibizumab immunogenicity demonstrates 5- to 20-fold greater potency due to higher affinity to VEGF molecules. The latest approved drug in the treatment of nAMD, aflibercept, consists of the VEGF-binding domains of human VEGFR1 and VEGFR2 fused to the Fc domain of human immunoglobulin-G1. Aflibercept acts as a decoy receptor binding not only VEGF but also placental growth factor (PLGF) [86–88].

In regard to aAMD, only antioxidant therapy in multiple forms is palliatively applied, which delays progression in 20–25% of eyes [75, 76, 89].

## The Hypoxia-inducible Factors

HIFs, the main regulators of hypoxia, are members of the basic helix-loop-helix Per-Arnt-Sim (bHLH-PAS) family. HIFs are composed of α and β subunits, where the latter is the constitutively expressed hydrocarbon receptor nuclear translocator (ARNT). Three isoforms have been identified for α subunits: HIF-1α, -2α and -3α [26, 27, 90, 91] with HIF-1α and HIF-2α being more prone for active responses to hypoxia. HIF-1α is widely expressed in all human normoxic and hypoxic tissues [92], while HIF-2α expression has been associated with physiologically hypoxic tissues [93] and spans 48% structure homology to HIF-1α [94]. The less characterized HIF-3α is expressed in adult thymus, lung, brain, heart, and kidney [95], and different splice variants of HIF-3α have opposite functions varying from activation to repression of hypoxia-inducible genes [91, 96].

Structurally, HIF-α subunits span at the N-terminus a basic helix-loop-helix (bHLH) and a Period-ARNT-Sim (PAS) domains responsible for DNA binding and dimerization to ARNT [97]. HIF-α transcription factors at the C-terminus contain two transactivation domains (TAD) which recruit coactivators [98, 99], and an inhibitory domain (ID) between the two TADs [100]. Oxygen-dependent protein regulation has been mapped to an internal oxygen-dependent degradation domain (ODD) [101]. In addition, two nuclear localization signals (C-terminal NLS and N-terminal NLS) have been identified, determining a near exclusive nuclear subcellular localization of HIF-α subunits [99, 102].

### Regulation of HIF expression

Interestingly, HIF-α subunits are constitutively expressed, yet protein levels and transcription activity are regulated by oxygen-dependent posttranslational modification (PTM), followed by epigenetic regulation of HIF-mediated transcription [103].

**Fig. 2:**
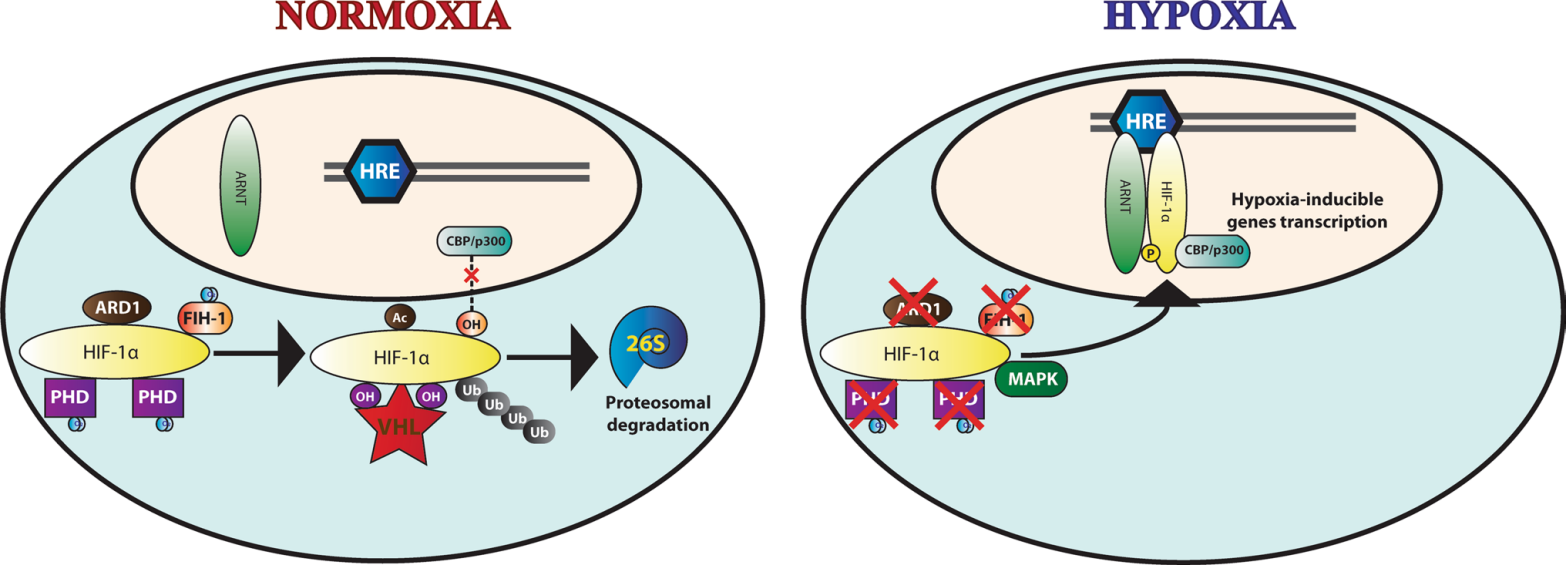
Regulatory mechanisms of HIF. **a** In normoxia, two prolines residues within the ODD of HIF-α are hydroxylated. Besides, a lysine acetylation by ARD1 ensues. These molecular reactions allow recognition of HIF-α by VHL ubiquitin ligase, which results in polyubiquitination of HIF-α and proteasome-mediated degradation. Simultaneously, an asparagine hydroxylation by FIH-1 blocks the interaction with CPB/p300 coactivators and critically impairs HIF-α transactivation. **b** In hypoxic conditions, PHD1-3, FIH-1 and ARD1 are rendered non-functional. HIF-α translocates to the nucleus and heterodimerizes with ARNT, forming the transcriptionally active HIF. The dimer is able to bind to HRE and initiate transcription of hypoxia-inducible genes. Moreover, MAPK phosphorylation of HIF-α enhances HIF transcriptional activity and recruitment of CPB/p300 coactivators

In normal oxygen conditions (Fig. 2), HIF-α is hydroxylated in two conserved prolines by a family of three oxygen-dependent orthologues of prolyl hydroxylase domain (PHD1-3) [104, 105]. Subsequent to hydroxylation of prolines 402 and 564 of HIF-1α (P405 and P531 in HIF-2α), the von Hippel–Lindau (pVHL), acting as the substrate-recognizing component of an E3 ubiquitin ligase complex, recognizes and polyubiquitinates HIF-α, resulting in 26S proteasome-mediated degradation of HIF-α proteins [106–109]. An additional oxygen-dependent asparaginyl hydroxylase factor inhibiting HIF-1α (FIH-1) regulates HIF transcriptional activation in normoxia. Hydroxylation of asparagine 803 of HIF-1α C-terminal TAD (N851 of HIF-2α) inhibits the binding of coactivators (CBP/p300) and renders HIF-α subunits transcriptionally inactive in normoxia [98, 99, 110, 111].

In addition, non-oxygen-dependent regulation of HIF-α has been attributed to lysine acetylation within the ODD by the ADP-ribosylation factor domain protein 1 (ARD1). Acetylation of lysine 532 promotes interaction between VHL and HIF-1α and stimulates HIF protein degradation. Despite oxygen-independent, PTM of HIF-α by acetylation is regulated by hypoxia-induced downregulation of ARD1 expression [112, 113].

In hypoxic conditions (Fig. 2), molecular oxygen is limited, the oxygen-dependent PHDs activity is compromised, and HIF remains unhydroxylated bypassing ubiquitin–proteasome degradation. Once stabilized and translocated to the nucleus, HIF-α heterodimerizes with ARNT, and the dimer binds to hypoxia-response elements (HRE; 5′RA/GCGTG3′) [114, 115], thus mediating upregulation of hypoxic genes [116]. Finally, phosphorylation of HIF-1α by p42/44 [117] and p38 [118] mitogen-activated protein kinases (MAPK) enhances heterodimerization and transcriptional activity of HIF, leading to the upregulation of hypoxic genes.

### Canonical HIF pathway

In hypoxia, the nuclear and transcriptionally active HIF heterodimer binds HRE, and the hypoxia-inducible genes are expressed [26, 119, 120]. Classically, the hypoxia-inducible genes are principally involved in cell differentiation, proliferation, and survival or apoptosis, cellular energy and metabolism, extracellular matrix (ECM) degradation and chemotaxis, and angiogenesis. HIF-mediated genes are involved also in a myriad of pathologies from stroke and ischemia, neovascular diseases, and several tumors and metastatic disease [26, 120].

In regard to tumor metastasis, HIFs are directly enrolled in tumor cellular growth by upregulating survival and proliferation genes, such as twist-related protein 1 (TWIST1) [121], integrins αvβ3, αvβ5 and αvβ6 [122], and several cadherins [123]. Concomitantly, HIF genes regulate ECM degradation and chemotaxis, modulating tumor metastasis directly. Matrixmetalloproteinases such as MMP2 [124] and MMP9 [125] together with urokinase-like plasminogen activator and its receptor (uPA; uPAR) and plasminogen activator inhibitor (PAI)-1 [126–128] are upregulated by HIFs in metastatic tumors. Moreover, the HIF-driven genes C–X–C motif chemokine receptor 4 (CXCR4) [129, 130] and C–C motif chemokine receptor 7 (CCR7) [131] contribute to metastasis guidance and dissemination of certain cancers.

Angiogenesis is regulated by HIFs, with VEGF and VEGFR major family as the principal intermediary [132–134]. In addition, calcitonin receptor-like receptor (CRLR) [135], stem cell factor (SCF) [136], and angiopoietin 2 (ANGPT2) [137] are overexpressed by HIFs and contribute to VEGF-independent angiogenesis.

Regarding cellular energy and metabolism, HIFs directly regulate the glycolytic shift from Krebs cycle to anaerobic pathways in hypoxia by upregulation of glucose transporters GLUT1 and GLUT3 [138, 139], glycolytic enzymes 6-phosphofructo-2-kinase/fructose-2,6-biphosphatase 3 (PFKFB3) [140], phosphoglycerate kinase 1 (PGK1) [141, 142], pyruvate kinase M2 (PKM2) [143], pyruvate dehydrogenase kinase 1 (PDK1) [144], lactate dehydrogenase A (LDHA) [114], c-Myc [145], and monocarboxylate transporter 4 (MCT4) [146].

On cellular differentiation processes, HIFs maintain cells undifferentiated by promoting the expression of octamer-binding transcription factor 4 (OCT-4), and stage-specific embryonic antigen-1 (SSEA-1) and SSEA-4 [147], therefore contributing the stemness of specific progenitor niches in adult tissues. In addition, HIFs contribute to de-differentiation of cancer cells by modulating the Notch-dependent epithelial-mesenchymal transition signal involved in the maintenance of undifferentiated cells [148, 149]. On the other hand, HIFs through erythropoietin and its receptor (EPO; EPOR) [150, 151] produce proliferation and differentiation of hematopoietic stem cells into red blood cells. In addition, HIF-1α upregulates the transcription factor SOX-9 expression, which participates in chondrogenesis [152].

Finally, HIFs have been reported to induce apoptosis via the p53 tumor-suppressor gene [153] and the B-cell lymphoma (*Bcl-2*) gene family—BNip3 [154] and Noxa [155]. On the other hand, HIF can contribute to prevent apoptosis by the upregulation of nucleophosmin (NPM) [156] and human urocortin 2 (*hUcn2*) [157].

## The role of HIF pathway in nAMD

Some tissues in the eye, including the cornea and the macula, are physiologically avascular and prone to hypoxia. In the eye, HIFs play specific physiologic roles in contributing to the avascularity: in the cornea, a splice variant of HIF-3α is responsible for blocking hypoxia-inducible neoangiogenesis during sleep [158], while in the foveal pit, HIFs contribute to the expression of VEGFR2 to sequester VEGF and impair vascularization into the macula [159]. In the particular case of the RPE layer, HIFs have been suggested to contribute to the apical secretion of pigment epithelium-derived factor (PEDF; antiangiogenic factor) and baso-lateral VEGF (angiogenic factor), thus simultaneously ensuring avascularity of the POS layer and vascular proximity from the choriocapillaries [27].

With regard to nAMD, genes encoding the canonical angiogenic factors VEGF [16, 132–134, 160, 161] and VEGFR [162, 163] have been widely associated with the development and progression of the disease. In addition, multiple molecular effectors have been associated with progression of nAMD [26] by contributing to: endothelial cell proliferation and recruitment of inflammatory cells—placental growth factor (PLGF) [164] and its receptor VEGFR1 [162], platelet-derived growth factor B (PDGF-B) [165] and its receptor PDGFRB [166], ANGPT-1, ANGPT-2 and their receptor Tie2 [161, 167], angiopoietin-like 4 (ANGPTL4) [168], stromal-derived growth factor-1 (SDF-1) [169] and its receptor CXCR4 [130]; ECM degradation—PAI-1 [126], and MMP2 and MMP9 [125]; vascular permeability and vasodilation—VEGF (particularly VEGF-A), VEGFR1, VEGFR2 [162, 163]; endothelial sprouting—ANGPT-2 [170]; and fibroplasia—transforming growth factor beta (TGF-β) [171].

Interestingly, all of the aforementioned factors, which directly contribute to nAMD, are HIF target genes [26, 27, 29, 69, 172, 173]. Drusen maculopathy and the age-related thickening of the BM together contribute to the increased hypoxic status of the RPE and thus lead to increased levels of HIF transcription factors. The expression of HIFs has been detected in choroidal neovascular membranes from clinical samples of nAMD patients [28, 29] and further corroborated to contribute to nAMD in mouse models of CNV [174, 175]. Moreover, HIFs also increase RPE apoptosis and autophagy, similarly to the choriocapillaries, which stimulates the endothelial proliferation [176–178] observed in nAMD.

## Addressing unmet needs in nAMD treatment

Nowadays, nAMD practitioners widely recur to anti-VEGF agents [79–82] for treatment, albeit with limitations or side effects: high probability of ocular infection, increased ocular pressure, cataract, vitreous hemorrhaging and retinal detachment; elevated price; transient (demands repeated intravitreal injections); limited recovery of patients’ visual acuity in a long period [179–182]. Some of these issues evolve from the use of mono-factor therapies, such as anti-VEGF drugs, to address a multifactorial, multi-genetic disease, as is the case of nAMD. In order to improve effectiveness, combination of treatment with other proangiogenic inhibitors as blocking PDGF ([183, 184], clinicaltrials.gov/NCT01940900), steroids, e.g., triamcinolone [185], or AINEs, such as ketorolac [186], and integrin antagonists [187] has been suggested as adjuvants to anti-VEGF regiments. Collectively, these novel therapeutic approaches indicate the need to simultaneously address a multitude of nAMD-associated factors. Considering its pivotal role in nAMD and master regulators of many factors associated with the disease, therapeutic targeting of HIFs reveals an attractive possibility.

### Pharmacological inhibition of HIF in nAMD

With HIFs as a central pathway in the pathogenesis of AMD [188–191], pharmacological inhibition of HIF would delimitate the production and secretion of many angiogenic factors and cytokines, in contrast to anti-VEGF drugs.

The inhibition of HIFs can be achieved indirectly by impairing pathways that mediate hypoxic responses, such as phosphoinositide 3-kinases/mammalian target of rapamycin (PI3K/mTOR) [192], topoisomerase I [193]/II [194], microtubules [195], Hsp90 [196], farnesyltransferase [197, 198], histone deacetylase [199], or thioredoxin 1 (TRX1) [200]. In animal models of ocular neovascular diseases, pharmacological inhibition of hypoxia-related pathways has displayed some success. Some of these drugs include cardiac glycosides as digoxin (inhibits p53 protein synthesis and target nuclear factor (NF)-κB; nuclear competitors of HIF) [201], anthracyclines as doxorubicin and daunorubicin (inhibits topoisomerase II, hence blocking the binding of HIF to DNA) [202], YC-1 (inactivates the upregulated PI3K/mTOR pathway, thus inducing degradation of HIF-1α) [203], and honokiol—which inactivates multiple pathways including NF-κB, mTOR, epidermal growth factor receptor (EGFR), signal transducer and activator of transcription 3 (STAT3), and caspase-mediated pathways, while also blocking the binding of HIFs to VEGF promoter and simultaneously decreasing VEGF secretion in RPE cells by HIF-independent effects on EGFR and STAT3 [204]. Expectedly, many of the indirect HIF inhibitors have shown considerable side effects both in vitro and in vivo studies, which implies a preferable direct inhibition approach. A multi-kinase inhibitor of uPAR co-receptor dimerization, UPARANT, has been demonstrated to indirectly inhibit HIF [205–208] with promising effects on systemic administration and unobserved side effects in animal models of ocular diseases, including nAMD [206].

Direct inhibition can affect different stages of HIFs, by reducing protein levels, interfering with dimerization, or binding to HREs. The first specific direct HIF inhibitor, echinomycin, has been reported to interfere with the HIF transcription factor binding to DNA [209], although reports of the effects of echinomycin on ocular cells or models are currently missing. Alternative direct inhibition of HIF has been achieved by overexpressing specific HIF modulators, such as PHD2 [210] or microRNAs [211]. Due to the intracellular nature, HIF modulator molecules must undergo gene therapy strategies.

## Gene therapy for the treatment of nAMD

Nowadays, a few inherited mono-genetic diseases can be treated by gene replacement therapies. Interestingly, gene therapy for a rare form of Leber congenital amaurosis (LCA) by the replacement of *Rpe65* gene—Luxturna—has been approved by the FDA [212].

The eye has been considered as an ideal organ for gene therapy-based transduction: it is compartmentalized and on the fringes of the immune system actions; the adult eye cell proliferation is limited and thus non-integrating vectors are feasible; easy access for direct treatment and evaluating effects; and need for small quantities of drug which correlate to neglectable-to-none systematic side effects. For AMD, gene therapy should be delivered in the macular subretinal space since RPE cells and photoreceptors are the principal targets of treatment [213]. Subretinal injections are becoming more standard with the currently approved ocular gene therapy treatment.

Generally, gene transfer can be achieved by viral and non-viral vectors [214], and the target gene can be replaced or inactivated. Viral vectors are more sustainable and effective than non-viral vectors, and thus preferable. Commonly used viral vectors in gene therapy models include recombinant adeno-associated virus (rAAV) vectors, adenovirus (Ad), and integrating-deficient lentivirus (IDLV) [215]. rAAV vectors have been the most effective for retinal gene therapy due to sustainable transduction of photoreceptors and RPE. Nonetheless, due to their small size, DNA capacity is limited to genes smaller than 4.7 kb [216]. In addition, rAAV vectors are non-integrating to the mammalian genome, thus displaying good safety profile as it is reported in multiple animal models, including large-eyed animals, such as dogs and primates [217]. It is noteworthy that minimal immune response to the rAAV vectors can occur [218, 219], although the use of specific viral serotypes, such as rAAV2, has demonstrated good tolerability in patients [220].

Ongoing clinical trials for gene therapies for the treatment of nAMD exploit protein-based and RNA interference (RNAi) antiangiogenic strategies. Considerable effort has been applied on protein-based gene therapy trial for nAMD, where the transduced cells overexpress angiostatic proteins in order to arrest CNV. As an example, subretinal rAAV-mediated gene transfer of the VEGF inhibitor—sVEGFR1—decreases choroidal vascularization in animal models by sequestration of VEGF and forming inactive heterodimers incapable of activating the VEGF receptors [221]. AAV vectors with other angiostatic factors, such as PEDF [222] and angiostatin [223], also have successfully arrested CNV in animal models. At present, clinical trials include Ad transducing PEDF protein [224], rAAV2 transducing sVEGFR1 (clinicaltrials.gov NCT01494805, NCT01024998, [221]), and the first lentiviral vector clinical trial, RetinoStat (clinicaltrials.gov NCT01301443, [225]), transducing two antiangiogenic genes: endostatin and angiostatin.

### Anti-HIF gene therapy in nAMD

Specifically targeting HIF transcription factors have been an attractive strategy for the treatment of the multifactorial nAMD [226–228]. Currently, only animal models of CNV-associated with nAMD have been addressed with anti-HIF gene therapies. Assessing HIFs directly appears to mitigate angiogenesis and inflammatory responses, both associated with nAMD disease initiation and progression.

Inhibition of HIFs by gene therapy constructs has most commonly been achieved by anti-HIF microRNAs (miRNA). The expression of miRNA-20b modulates VEGF by targeting HIF-1α and STAT3 in MCF-7 breast cancer cells [229, 230] and has been suggested as a putative candidate for nAMD gene therapy [26, 27]. Nevertheless, miRNAs have been suggested to display considerable unspecificity by targeting multiple pathways; therefore, the use of HIF-specific RNAi has been reported beneficial in AMD models [211].

Use of protein-based gene therapy strategies for the treatment of CNV-associated with nAMD has been less explored, partly due to the lack of RPE- and HIF-specific modulators of the hypoxia pathway. Gene transfer of PHD2 in vivo resulted in the mitigation of HIF-mediated angiogenesis in a mouse model of nAMD [210] by reducing several nAMD-associated angiogenic factors and cytokines. PHD2 has been suggested as the ideal candidate for targeting HIF in RPE cells and has considerable HIF-selectivity in hypoxia [231] rendering it a putative candidate for anti-HIF gene therapy treatments of nAMD.

## Future perspectives

Treatment of multifactorial diseases, such as nAMD, presents an immense clinical challenge. Despite prevalent use and significant success, current treatments for patients afflicted with nAMD by the administration of anti-VEGF drugs are far from optimal. Treatments must be administered on routine basis with substantial cost for health care systems and associated risks for the patients. Development of sustainable treatments could considerably improve nAMD therapies.

Gene therapy presents a possibility of sustainability when compared to pharmacological or surgical treatments, since the treatment is localized to the target cells, sustained with one dose, and regulated through gene-construct engineering. The regulation of gene expression is desirable to minimize side effects [226, 232]. The use of tissue- and cell-specific promoters has increased targeted expression of therapeutic genes in gene therapy. In nAMD, the involvement of RPE cells is pivotal, and RPE-specific promotors, such as p*Rpe65* (encoding RPE65 protein) and p*VMD2* (promoter for the vitelliform macular dystrophy 2, encoding bestrophin-1 protein), grant expression of therapeutic genes specifically in RPE cells. Albeit non-integrative viral vectors are used for gene therapy in ophthalmology, the post-mitotic character of adult RPE and photoreceptors results in long-term expression of transduced genes. Despite the attractiveness of sustainable treatments for nAMD, in opposition to the need for routine injections currently applied with anti-VEGF drugs, the risk of long-term secondary events of the therapeutic gene should be considered. Engineering of inducible cell-specific promoters to the administration of exogenous genes for the treatment of nAMD has been proposed [233]. Combining tissue-specific promoters (p*Rpe65*) with HRE regulatory elements, hypoxia-mediated spatial and temporal regulation of angiostatic proteins can be achieved to mitigate CNV in mouse models of nAMD [234].

The subretinal injection routinely used to deliver gene therapy to RPE cells requires a vitrectomy to hamper retinal detachments, which could result in surgical complications. Present intravitreal transduction of gene therapy vectors most commonly is limited to the inner layers of the retina [235, 236], yet modified adeno-associated virus (AAV) capsids have been reported to transduce both photoreceptor and RPE cells in non-human primates [237]. Development of novel mutant virus packaging capsids of different serotype will certainly improve gene expression kinetics and cellular tropism for the future of gene therapies [238–240].

## Conclusion

Anti-HIF therapies have demonstrated considerable improvement in models of nAMD when compared to anti-VEGF drugs. Targeting a transcription factor can present its own challenges, yet advances on gene therapy strategies have paved bright conceptual avenues for future anti-HIF sustainable and long-term treatments for nAMD patients.
